# Mechanical and Tribological Characterization of WC-Co and WC-AISI 304 Composites by a Newly Developed Equipment

**DOI:** 10.3390/ma15031187

**Published:** 2022-02-04

**Authors:** Luís Vilhena, Bruno Domingues, Cristina Fernandes, Ana Senos, Amílcar Ramalho

**Affiliations:** 1 Department of Mechanical Engineering, Centre for Mechanical Engineering, Materials and Processes (CEMMPRE), University Coimbra, 3004-516 Coimbra, Portugal; bmarques_6@hotmail.com (B.D.); amilcar.ramalho@dem.uc.pt (A.R.); 2 Department of Materials and Ceramic Engineering/CICECO, University of Aveiro, 3810-193 Aveiro, Portugal; cmfernandes@ua.pt (C.F.); anamor@ua.pt (A.S.)

**Keywords:** hardmetals, cemented carbides, WC-composites, AISI 304 binder, Pin-on-Disc, friction, wear

## Abstract

Tungsten carbide-based composites are, in many cases, the materials of choice in applications requiring high wear resistance. In the present research work, the mechanical characterization of the WC-Co and WC-AISI 304 composites was carried out, with evaluation of the hardness and fracture toughness and tribological characterization of the composites that included the study of friction and wear rate coefficient through unlubricated sliding tests according to the Pin-on-Disc test method. It was possible to correlate the effect of the different binding phases on the mechanical and tribological properties of WC-based composites, and it can be concluded that the system composed by the tribological pair WC-AISI304/100Cr6 was the one that showed the lowest coefficient of friction while the tribological pair WC-Co/Al_2_O_3_ was the one that showed the lowest wear rate coefficient.

## 1. Introduction

Tungsten carbide (WC) based composites, also known as cemented carbides or hardmetals, stand out for their mechanical properties, but mainly for the excellent wear resistance they offer [1,2,3]. Cemented carbides are typically made up of a high-volume fraction of hexagonal tungsten carbide particles embedded in a cobalt metal matrix [4]. The tungsten carbide particles are characterized by high hardness and brittleness while the metallic matrix, called binder, gives the composite strength and ductility [5]. The combination of two such distinct phases permit the obtainment of a material with very specific properties, combining high hardness, good fracture toughness, high resistance to wear and corrosion and the ability to operate in environments with high pressures, high loads, and high temperatures [3,4,6].

WC-based composites are mainly used when wear resistance is a key factor. The applicability of these materials is vast and can be used, for example, in tools for cutting and forming metals, components for the mining and mineralogical industry, drilling tools for the oil and gas extraction industry, military components and even in jewelry [4,7,8].

**Figure 1:**
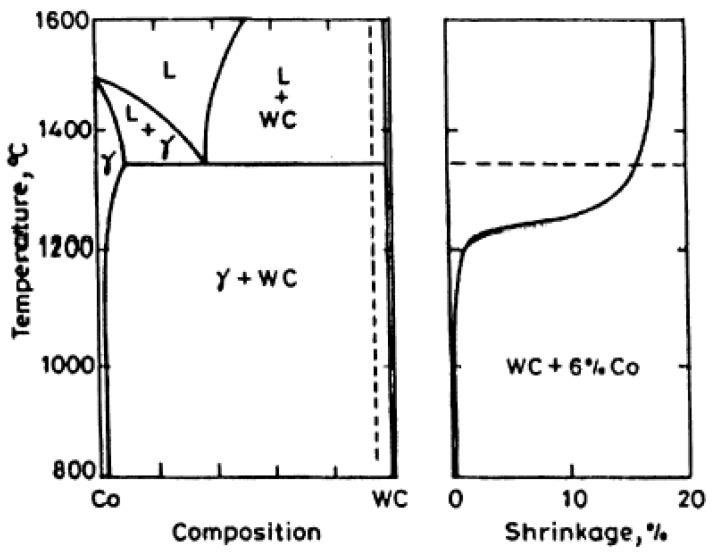
WC-Co pseudo-binary diagram (left side) and shrinkage during the WC-6Co sintering phase (right side) [5].

Composites based on tungsten carbide, such as WC-Co, are produced by liquid phase sintering at temperatures between 1370 °C and 1425 °C, below both the melting temperature of the WC (2800 °C) and the pure cobalt (1495 °C) [5]. This method comprises four steps. In the first stage, the composite retracts due to the reduction of oxides and the degassing of the binder and impurities. In the second stage, the temperature rises and starts the solid-state sintering process. Porosity decreases and densification occurs due to the rearrangement of particles and solid-state diffusion mechanisms [9]. The temperature continues to rise and promotes the formation of a liquid phase above the eutectic temperature [10]. The third stage begins, consisting of sintering in the liquid phase, in which the WC grains are dissolved in the binder until saturation. During this stage, and accompanying the densification, smaller grains are preferably dissolved and those of a larger size tend to grow. Figure 1 shows, on the left side, the WC-Co pseudo-binary diagram and, on the right side, the shrinkage verified during the WC-6Co sintering phase. In the last stage, consisting of cooling, the binder solidifies and there may be reprecipitation of W and C contributing to an increase in grain size [9].

**Figure 2:**
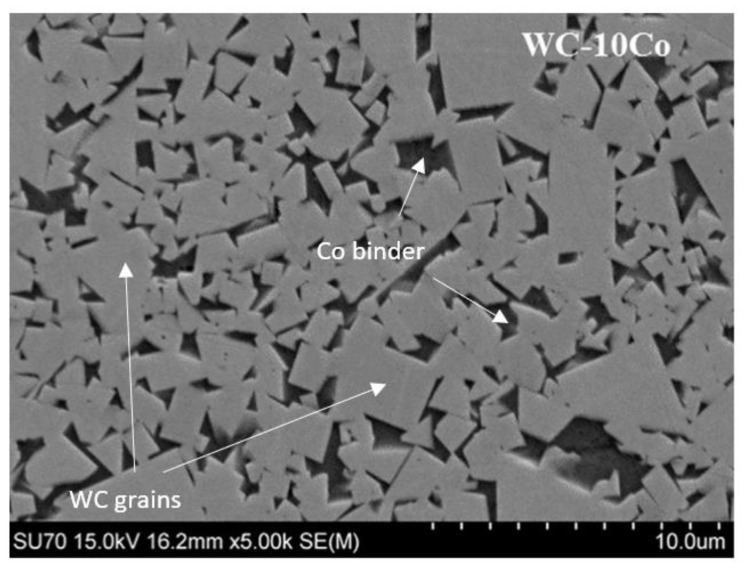
SEM micrograph of the WC-10Co composite.

Cobalt has been the metal of choice in WC-based composites since 1926 after the production of the first hard metal, WIDIA-N, composed of 6% by weight of cobalt. This metallic element has excellent characteristics, being used as a binder in about 90% of all manufactured cemented carbides, with compositions ranging from 3 to 25 wt.%. One of the main advantages in using cobalt as a binder in WC-based composites is the high densification it promotes due to the high wettability of the WC particles. Figure 2 represents the typical microstructure of a WC-based composite with a cobalt binder. The WC particles (light gray areas) assume a prismatic shape and have a uniform distribution through the cobalt binder (dark gray areas).

Despite all the advantages that cobalt presents as a binding phase, there are also several problems associated with its use. Currently, cobalt-based tungsten carbide composites are included in group 2A of the International Agency for Research on Cancer, IARC, identified as potentially carcinogenic [11]. Cobalt, on the other hand, is part of IARC group 1, classified as a carcinogen for humans. According to the European Chemicals Agency, ECHA [12], the element cobalt, in addition to being carcinogenic, is suspected of being mutagenic, which can affect fertility. It can also cause allergic skin reactions and breathing difficulties if inhaled, as well as long-lasting effects in the aquatic environment. In addition to the various metallurgical applications, more than half of the cobalt produced is destined for the manufacture of lithium-ion batteries. This type of batteries is used in electronic equipment such as laptops, smartphones and electric vehicles [13]. The sharp demand for lithium-ion batteries has caused an increase in demand for cobalt, which results into an increase in its cost.

Considering the reasons highlighted, it is necessary to find new solutions that allow the replacement of the cobalt binder by a more economical alternative, less toxic and with equal or superior properties. The alternative with the greatest potential is the total or partial replacement of cobalt by other transition metals such as Fe, Cr and Ni [14,15,16,17,18,19,20,21,22,23,24]. The traditional WC-Co has also some limitations with respect to corrosion resistance and in that sense the use of Fe and Ni based binders has been the target of research to improve this property in carbides [25,26,27].

Studies carried out with WC-Ni-Fe and WC-AISI 304 demonstrated superior corrosion resistance if compared to the traditional WC-Co composites [28,29]. WC-AISI 304 is a composite based on tungsten carbide with a binder phase in stainless steel AISI 304, having as main constituents Ni, Fe and Cr. This material is part of the WC-SS (Stainless Steel) composites family. In a study by B.J. Marques et al. [29] on the application of the stainless-steel binder AISI304 in hard metal, with contents varying between 6 and 15 wt.%, it was found that the binder showed good wettability during the sintering phase, at temperatures between 1440 and 1520 °C, being possible to obtain, after hot isostatic pressing, high relative densities between 96–99%. It should also be noted that the study indicates an increase in hardness compared to WC-Co, with fracture toughness being preserved. Similar results were obtained by our research team [4,5,6].

Of the various types of wear to which these materials are subject, the one that most needs investigation is the sliding wear. Although there are already several studies on sliding wear in WC-based composites with cobalt binding phase, there are, to date, no comparative studies between WC-Co and WC-AISI 304 composites that evaluate non-lubricated sliding wear and their mechanisms. The aim of the present study is the mechanical and tribological characterization of the WC-based composites with binder phase in AISI 304 stainless steel (WC-AISI 304) and its comparison with the traditionally used binder (WC-Co). The technique used for experiments was Pin-on-Disc unidirectional sliding in a tribometer developed by our team, using two different counter bodies of different materials, 100Cr6 and Al_2_O_3_.

## 2. Materials and Methods

### 2.1. Specimens

**Figure 3:**
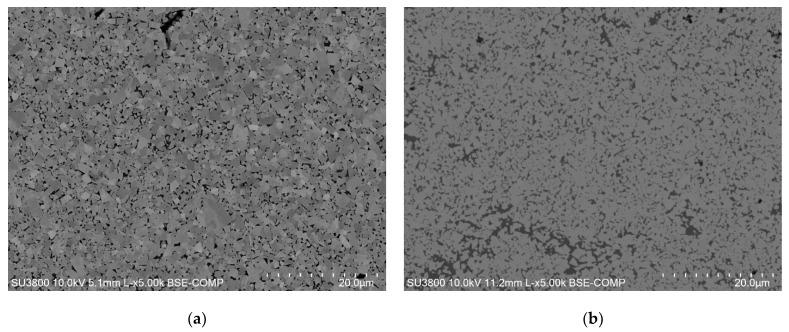
SEM micrographs of the: (**a**) WC-10Co composite and, (**b**) WC-AISI 304 composite.

To carry out the experimental tests, two types of tungsten carbide-based composites were selected, WC-Co and WC-AISI 304, both supplied by the company specialized in hard metal, DURIT-Metalurgia Portuguesa do Tungsténio (Albergaria-a-Velha, Portugal). The WC-Co composite corresponds to the traditional carbide, while the WC-AISI 304 has been investigated as a potential alternative, as a way of replacing cobalt in these materials. The samples under study have a disk shape and their compositions are shown in Table 1 and the microstructures are shown in Figure 3.

The present research work is more focused on the tribological characterization (unidirectional sliding wear) and not so much on processing and microstructural characterization. However, previous XRD analysis of the sintered specimens performed by our team can be seen elsewhere [4] and indicates the presence of WC as major phase, η-phase (Fe3W3C) and Fe(γ) for WC–10 AISI 304 with a densification of approximately 99.7%.

The WC-Co and WC-AISI 304 specimens were subjected to a surface polishing process using Struers LaboPol-5 equipment using a 6 µm diamond paste. After sample preparation, surface roughness was evaluated. For this, three tests were performed on each prepared face, with a “mirror-like” finishing, using the Mitutoyo Suftest 402 instrument. All specimens showed an average R_a_ roughness parameter equal to or less than 0.02 µm. To study the influence of the material of the counter body in unidirectional sliding tests, two materials with different characteristics were selected. One of metallic type (100Cr6) and a second of ceramic type (Al_2_O_3_). 100Cr6 steel is a low martensitic alloy steel, with high hardness and wear resistance. It has a hardness between 60 to 66 HRC-Rockwell C scale [30]. Alumina-Al_2_O_3_ is a ceramic material also characterized by high hardness and high resistance to wear. According to the literature, hardness is usually in the range between 15 to 19 GPa [31]. In order to evaluate the mechanical properties of the WC-Co and WC-AISI 304 specimens, their hardness and fracture toughness were determined. The hardness was determined using the Vickers indentation test using the Zwick/Roell ZHU equipment. The tests were carried out with a load of 30 Kg (294 N), applied for 15 s (HV_30_). To determine the fracture toughness, the Palmqvist method was used. This method allows to quantify the fracture toughness using the K_IC_ factor based on the length of the cracks formed during the Vickers indentation test. This factor can be calculated using the Equation (1) suggested by Shetty et al. [32].

(1) $$K_{IC}=0.0889\times \sqrt{H}\sqrt{\frac{P}{\Sigma l}}$$

where *H* represents the hardness in Pascal (Pa), *P* the load in Newton (N) and 𝛴*l* the sum of the crack lengths, expressed in meters (m). In the measurement of hardness and fracture toughness, five values were obtained for each composite, with the results shown below being their average and standard deviation.

### 2.2. Tribological Testing

**Figure 4:**
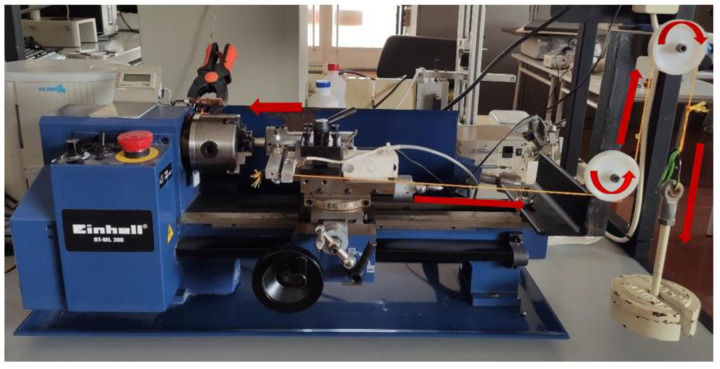
Pin-on-Disc Tribometer used in tribological tests (the red arrows indicate the direction of application of the force).

To carry out the experimental work, a tribometer built in our laboratory and based on a lathe was used, as shown in Figure 4. This equipment was designed for a Pin-on-Disc configuration, which allows the execution of wear tests by unidirectional sliding, non-lubricated, according to the requirements established by the ASTM G99 standard.

**Figure 5:**
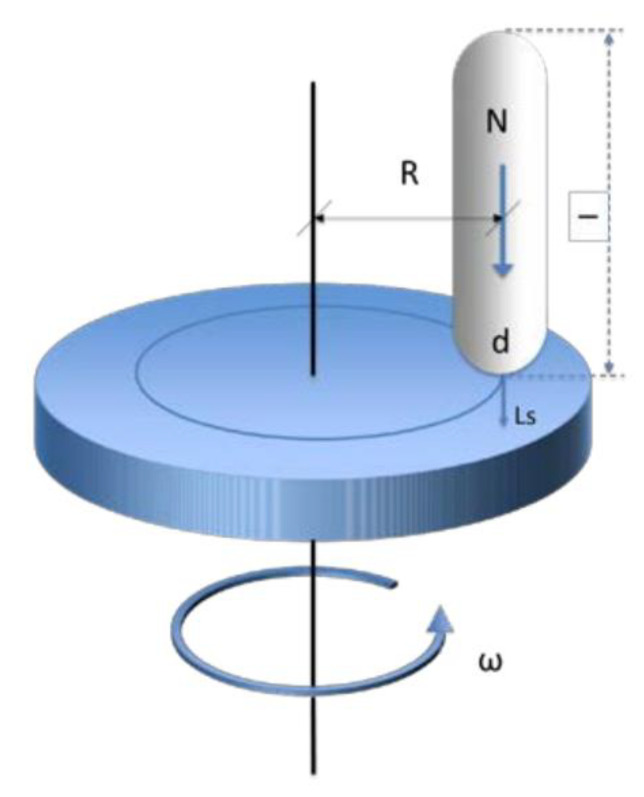
Diagram of the Pin-on-Disc test method [33].

In the Pin-on-Disc test method, a disc rotating around its axis is brought into contact with a stationary pin, following a unidirectional displacement in which a normal force is applied. Figure 5 outlines this process.

**Figure 6:**
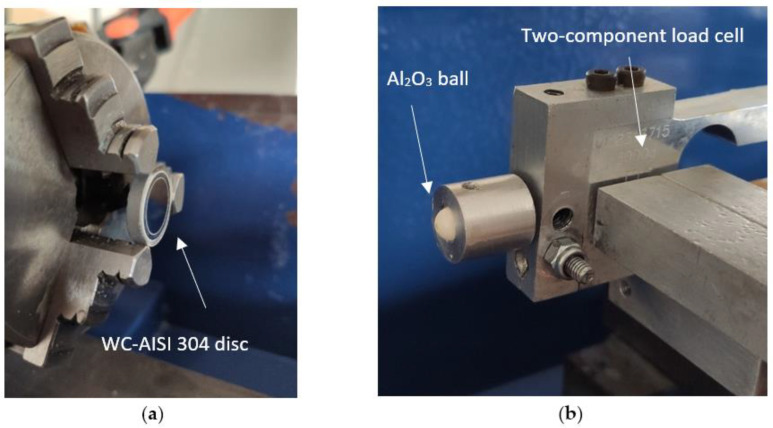
Mechanism for fixing the disks to the tribometer (**a**). Mounting the conical support for fixing the counter body on the tribometer (**b**).

In this study, specimens of WC-Co and WC-AISI 304 were used as discs, while the 100Cr6 and Al_2_O_3_ spheres corresponded to the pin, also designated as counter body. The disks are assembled using jaws on the rotating head of the equipment that allows the disks to be fixed during the tests, as shown in Figure 6a. For the case of the spheres, they are fitted in a conical support and in turn, they are fixed in the tribometer using a screw. The mechanism for fixing the counter body is illustrated in Figure 6b.

The tribometer was designed and built in our laboratory thus allows a set of parameters to be adjusted so that it is possible to define the test conditions, such as: speed of rotation of the disc (rpm), radius of the wear track as well as the normal applied load to the sphere that is secured by a suspended weight connected to the mechanism for fixing the counter body by means of a cable, as shown in Figure 4. This assembly ensures that the load is constant throughout the test.

To monitor friction during tribological tests, a controller was associated with the tribometer and connected to a computer where an interface was developed in the LabView 8 software. A tangential load cell installed in the equipment provides the program with the tangential force value in each instant. From the normal force entered by the user, constant throughout the test, and the data collected by the tangential load cell, the software calculates the instantaneous friction coefficient and presents the graph of the frictional force as a function of the elapsed time. All the equations used during this research work are presented and explained in Appendix A of the present manuscript.

For the present study, a linear sliding speed of 0.25 m/s and a normal applied load of 20 N were defined and was maintained constant for all tests. Four tribological systems were evaluated. These systems are formed by the tungsten carbide-based composites WC-Co and WC-AISI 304 combined with the counter bodies 100Cr6 and Al_2_O_3_. Table 2 identifies the referred systems.

The experimental procedure started with cleaning the specimens and counter body, using ultrasound equipment. Then they were mounted on the respective supports of the pin and disk tribometer. The equipment’s rotation speed was defined and adjusted, as well as the distance between the centre of rotation of the disk and the centre of the sphere to guarantee the desired linear sliding speed. The counter-body was then pressed against the disc and the weight that ensures the normal force applied was hung. The duration of each test was previously calculated for a certain number of revolutions based on the rotation speed of the equipment and the distance between the centre of rotation of the disk and the centre of the sphere.

To assess the wear mechanism, as well as particles observation collected from its surface, the wear track of tungsten carbide-based composite discs was investigated using a Philips XL30 and a Hitachi SU-70 and a SU-3800 scanning electron microscope. In the case of particle samples, it was necessary to coat them in gold/platinum using cathodic deposition techniques.

## 3. Results and Discussion

Following the Vickers hardness and fracture toughness tests using the Palmqvist method for WC-Co and WC-AISI 304 specimens, the results obtained are shown in Table 3. 

The first phase of discussion is centered on the mechanical characterization of the WC-Co and WC-AISI 304 composites. Through the Vickers test, the HV_30_ hardness (kgf/mm^2^) of 1491 ± 36 was determined for the WC-Co and 1542 ± 18 for WC-AISI 304. Subsequently, using the Palmqvist test, the fracture toughness, K_IC_ (MPa·m^1/2^), of 9.7 ± 1.1 for WC-Co and 7.8 ± 0.3 for WC-AISI 304 was determined.

L.M. Vilhena et al. [6] characterized the abrasive wear resistance of WC-Co and WC-AISI 304 composites, where they measured their mechanical properties. Regarding the values calculated in this work for the mechanical properties of WC-Co, when compared with results obtained by L.M. Vilhena et al. [6] for the same material, it was found that the hardness was about 15% higher and the fracture toughness 34% lower. Regarding the WC-AISI 304 composite, also in comparison with the results acquired by L.M. Vilhena et al. [6], there was a great proximity, having obtained values for this hardness and fracture toughness 6 and 2% lower, respectively. The values related to the mechanical properties agree with those proposed by L.M. Vilhena et al. [6], with the largest difference corresponding to WC-Co fracture toughness. The differences found may be based on variations in composition, microstructure, or grain size between the various composites. When comparing the values of the properties of the two composites under study, the tendency corroborated that the increase in hardness implies a decrease in fracture toughness.

### 3.1. Friction Behavior 

Table 4 presents the experimental conditions of the four tests performed for the tribological system consisting of the WC-Co/100Cr6 tribological pair. For all other systems the same experimental conditions were followed.

**Figure 7:**
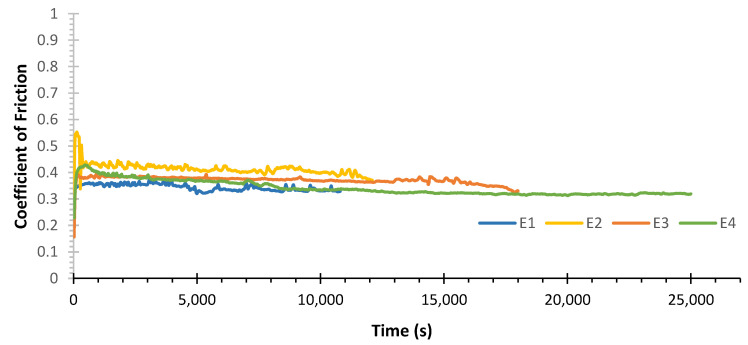
Coefficient of Friction (COF) curves for the tests performed for the system WC-Co/100Cr6.

In Figure 7, the friction curves corresponding to the tests performed for the system WC-Co/100Cr6 can be observed.

**Figure 8:**
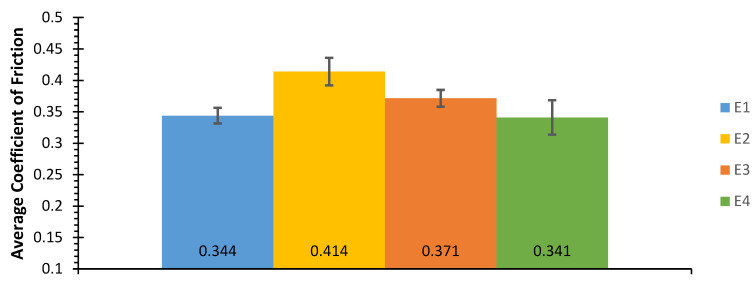
Average coefficient of friction and variation of the average coefficient of friction for the system WC-Co/100Cr6.

Analysing the friction curves shown in Figure 7, it is possible to observe a decrease in the friction coefficient over time for all curves, being more evident in the case of the E4 test. It is also verified that the curve of the E2 test showed higher COF then the other curves in its entire length. Figure 8 shows the average coefficient of friction values for the system WC-Co/100Cr6, determined using the arithmetic mean of the set of instantaneous coefficients of friction for each tribological test.

Analysing Figure 8, it can be concluded that the average coefficient of friction of the E1 and E4 tests are very close, corresponding, respectively, to the tests with the smallest and largest sliding distance of the system WC-Co/100Cr6. In the E2 and E3 tests, with sliding distances between the tests E1 and E4, there was an increase in the average coefficients of friction. Thus, these results are not indicative of the influence of the sliding distance on the average coefficient of friction. The lowest average coefficient of friction occurs for test E4 with a value of 0.341. In contrast, in the test E2, the highest mean coefficient of friction, corresponding to 0.414, is verified. The variation of the average coefficient of friction is a parameter of great importance that must be determined to complement and fit the average coefficient of friction of each test. Therefore, the average coefficients of friction and the respective variations are shown in Table 5.

**Figure 9:**
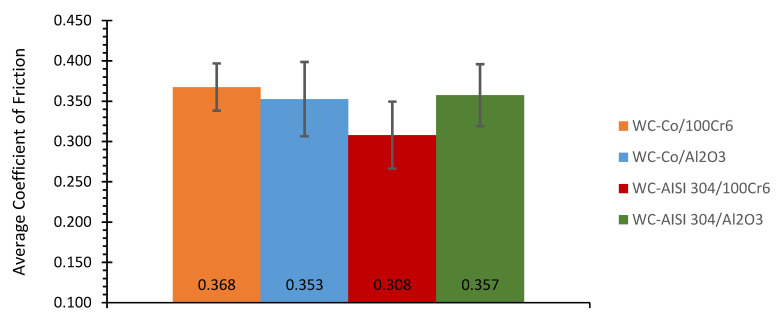
Average coefficient of friction and variation of the average coefficient of friction for the systems: WC-Co/100Cr6, WC-Co/Al_2_O_3_, WC-AISI 304/100Cr6 and WC-AISI 304/Al_2_O_3_.

The smallest variation in the coefficient of friction occurs in test E1 with the value of 0.012 and, in contrast, the largest variation for test E4 corresponding to 0.027. As shown for the system WC-Co/100Cr6, the same procedures were performed for the other systems, WC-Co/Al_2_O_3_, WC-AISI 304/100Cr6 and WC-AISI 304/Al_2_O_3_ and in Figure 9 the average coefficients of friction corresponding to all systems are shown. All the friction curves correspondent to the other systems can be seen in the Appendix B.

When analysing Figure 9, it can be seen that the lowest average coefficient of friction corresponds to the WC-AISI 304/100Cr6 system with a value of 0.308. The coefficients of friction corresponding to systems WC-Co/Al_2_O_3_ and WC-AISI 304/Al_2_O_3_ are 15 and 16% higher, respectively. System WC-Co/100Cr6 shows the highest average coefficient of friction with a value of 0.368, 19% higher than System WC-AISI 304/100Cr6. Table 6 illustrates the average coefficients of friction for each system as well as the respective variations.

In system WC-Co/100Cr6 there is the smallest variation in the coefficient of friction, setting at 0.029. In system WC-Co/Al_2_O_3_, there is the greatest variation in the coefficient of friction with the value of 0.046. Friction can be described as a force that opposes relative movement between bodies. This phenomenon is believed to be the result of three processes: adhesion, plowing and plastic deformation of surface asperities. Although the various materials under study have a good surface finish and therefore low roughness, the wear process causes an increase in the surface roughness and thus the development of friction.

For all friction curves, a rapid increase in the coefficient of friction was visible in the first meters of sliding. This phenomenon corresponds to the running-in regime, in which polishing reduce surface asperities through plastic deformation. However, after the running-in regime, two distinct behaviors were observed. Some curves tended to stabilize and slightly decrease the friction coefficient over time, while others showed multiple peaks or undulating behavior over time.

When relating the behavior of the friction curves to the type of composite used, a trend is verified. The curves corresponding to the tribological tests in which the WC-Co was used showed a more stable behavior compared to the friction curves belonging to the WC-AISI 304. 

### 3.2. Wear Behaviour

**Figure 10:**
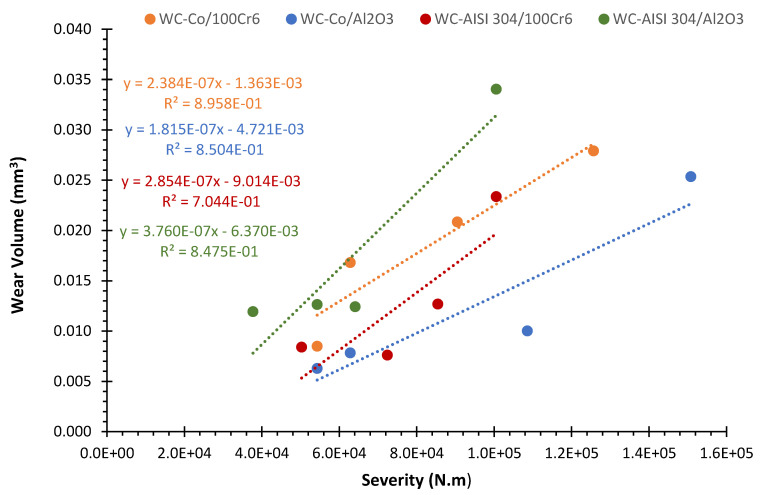
Disc wear coefficients for the systems: WC-Co/100Cr6, WC-Co/Al_2_O_3_, WC-AISI 304/100Cr6 and WC-AISI 304/Al_2_O_3_.

The wear coefficient allows to characterize the wear resistance of a given tribosystem. Figure 10 shows the trendlines of the four systems from which the respective wear coefficients are determined.

**Figure 11:**
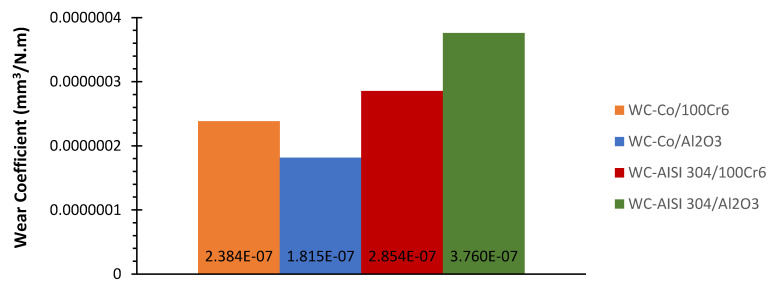
Disc wear coefficients for the systems: WC-Co/100Cr6, WC-Co/Al_2_O_3_, WC-AISI 304/100Cr6 and WC-AISI 304/Al_2_O_3_.

To facilitate the comparison, the wear coefficients obtained for the discs of the various systems under study are shown in the graph of Figure 11.

In a first analysis of the discs wear coefficients, it appears that they have the same order of magnitude. The lowest wear coefficient belongs to system WC-Co/Al_2_O_3_ with the value of 1.815 × 10^−7^ mm^3^/N.m. Following, are the wear coefficients of systems WC-Co/100Cr6 and WC-AISI 304/100Cr6, which are 31 and 57% higher, respectively. The highest wear coefficient corresponds to the system WC-AISI 304/Al_2_O_3_, with 3.760 × 10^−7^ mm^3^/N.m. This value reflects an increase of 107% when compared to the system WC-Co/Al_2_O_3_. 

Table 7 shows the wear coefficients of the counter bodies (K_cb_) and the respective percentage of increase in relation to the lowest coefficient found, corresponding to the system WC-Co/Al_2_O_3_.

The lower wear coefficient of the counter bodies is shown in system WC-Co/Al_2_O_3_. The coefficients of systems WC-Co/100Cr6 and WC-AISI 304/Al_2_O_3_, compared to the previous one, are 1.2 X and 8 X, respectively, higher. System WC-AISI 304/100Cr6 has, by an enormous margin, the highest coefficient, which is 98 X higher when compared to system WC-Co/Al_2_O_3_. 

To minimize the number of variables, the tribological tests were carried out according to a constant sliding speed and applied normal load and focused the study on the WC-Co and WC-AISI 304 composites as well as on the influence of the material of the counter-body. The results of the various tribological tests indicate that the wear volume of the sample discs tends to increase linearly with the increase in severity, in accordance with the prediction of Czichos relation expressed in Equation (A8) of the Appendix A. By analyzing Figure 10, this can be verified for all systems involved, through monotonic growth. However, Figure 10 also shows that, essentially for the WC-Co/Al_2_O_3_ and WC-AISI 304/Al_2_O_3_ systems, after a certain severity value (between the third and the fourth point), there is an abrupt increase in the wear value (the order of magnitude jumps from 1.00 × 10^−2^ mm^3^ to 2.534 × 10^−2^ mm^3^ and from 1.242 × 10^−2^ mm^3^ to 3.403 × 10^−2^ mm^3^, respectively, for the WC-Co/Al_2_O_3_ and WC-AISI 304/Al_2_O_3_ system, which is a behavior that represents a transition between a moderate and severe wear regime. For the WC-Co/Al_2_O_3_ system, the regime change was noted between the severity values of 1.09 × 10^5^ N.m and 1.51 × 10^5^ N.m. In the case of the system WC-AISI 304/Al_2_O_3_, this same type of sudden variation was observed for lower severity values, between 6.41 × 10^−4^ N.m and 1.01 × 10^5^ N.m.

This behavior is also evident in the case of counter-bodies. Regarding the WC-Co/Al_2_O_3_ tribological pair, the lowest wear coefficient of the disc and of the counter-body was observed. The second lowest wear coefficient, both for the disc and for the counter-body, concerns the system WC-Co/100Cr6. However, analyzing the systems, which use the composite WC-AISI 304, another scenario is verified. The use of the alumina counter-body in system WC-AISI 304/Al_2_O_3_ implies a higher wear coefficient of the disk compared to system WC-AISI 304/100Cr6 where 100Cr6 was used. In systems WC-AISI 304/100Cr6 and WC-AISI 304/Al_2_O_3_, it can also be observed that the lowest wear coefficient of the disc corresponds to the highest wear coefficient of the counter-body and vice versa. Based on these results, it can be concluded that the wear coefficient depends on the tribological pair used. Overall, the WC-Co composite showed better performance in terms of wear, while the WC-AISI 304 composite, which presents itself as an alternative to traditional WC-based composites, in its best scenario showed a 57% higher wear coefficient compared to the best performance of WC-Co.

**Figure 12:**
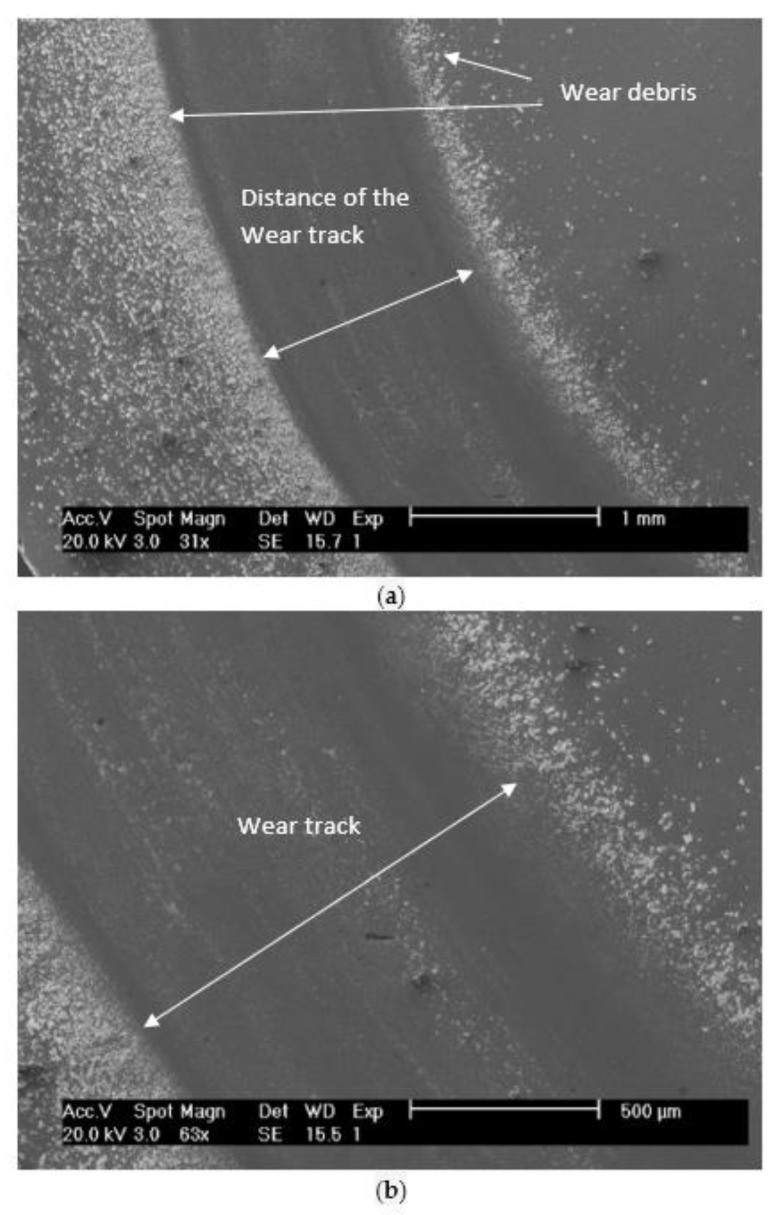
SEM micrographs of the wear track and debris resulting from the wear of system WC-AISI 304/Al_2_O_3_. According to a magnification of 31× (**a**), according to a magnification of 63× (**b**).

SEM analysis performed on the unclean disc after performing the tribological test, belonging to the system WC-AISI 304/Al_2_O_3_, allowed to observe its wear track, as well as the deposition of debris from sliding wear. In Figure 12, the wear track can be seen according to two magnifications. There is uniform deposition of worn material along the periphery of the wear track. This non-compacted worn material is characterized by its fine granulometry. The accumulated debris at the periphery of the wear track has no influence on wear as it is located outside the surface where the sliding occurs.

**Figure 13:**
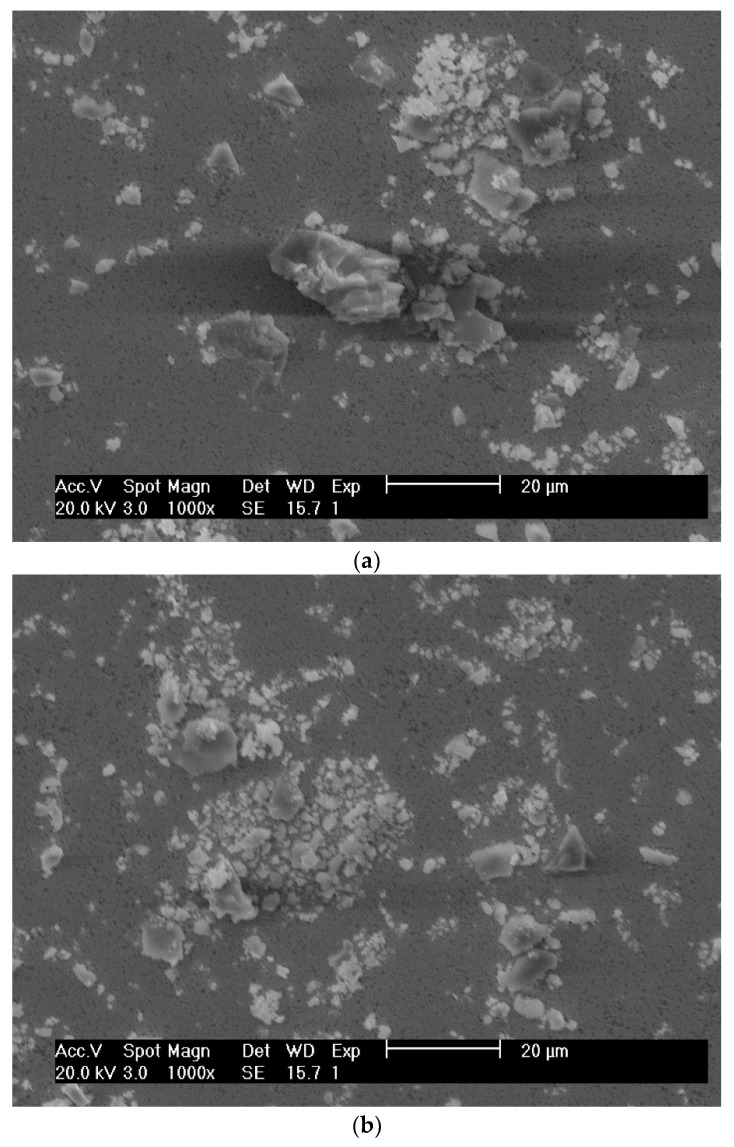
SEM micrographs of debris resulting from the wear of system WC-AISI 304/Al_2_O_3_, corresponding to two different locations on the periphery of the same wear track: (**a**) larger debris, (**b**) smaller debris.

In Figure 13, debris resulting from the interaction between the sliding surfaces is presented in detail, corresponding to two different locations on the periphery of the same wear track. It is possible to notice that the worn material assumes an irregular and angular shape with different dimensions.

**Figure 14:**
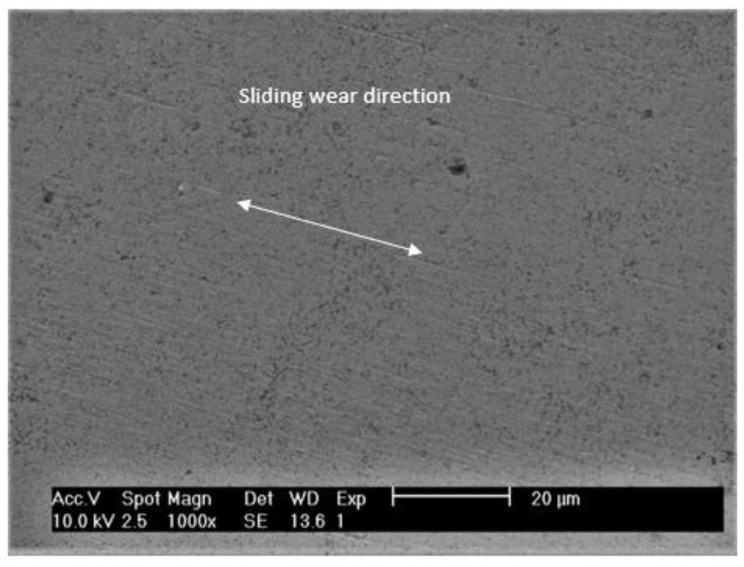
SEM micrograph showing the preferential direction of striations or ploughing that are parallel to the sliding direction for the system WC-AISI 304/Al_2_O_3_.

From the SEM analysis, it was possible to observe the preferential direction of striations or ploughing on the wear track, that is parallel to the sliding direction and are illustrated in Figure 14.

**Figure 15:**
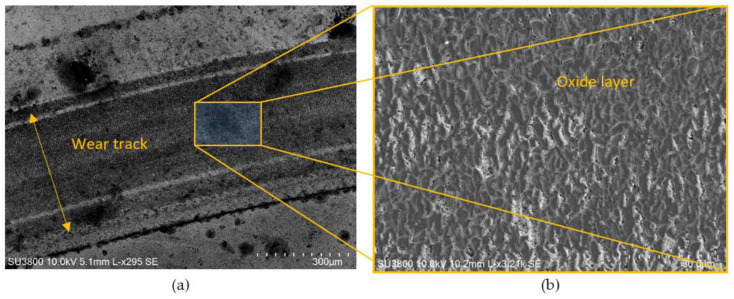
SEM micrographs of the wear track for the system WC-Co/Al_2_O_3_ according to a magnification of: (**a**) 925× and, (**b**), 3.21K×.

**Figure 16:**
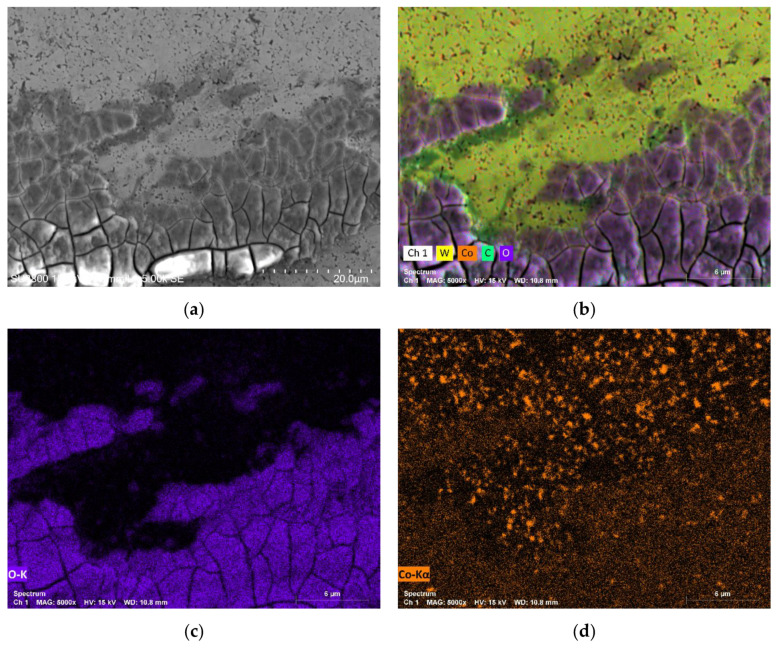

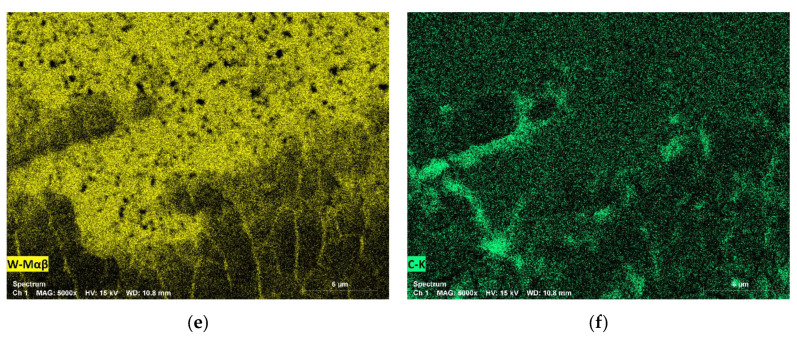
SEM micrograph and EDS analysis for the system WC-Co/Al_2_O_3_ showing the chemical composition in the border between the wear track with oxides formation and the unworn surface: (**a**) SEM with 5kx magnification; (**b**) Map with the spectrum of all chemical elements; (**c**) Spectrum of Oxygen; (**d**) Spectrum of Cobalt; (**e**) Spectrum of Tungsten; (**f**) Spectrum of Carbon.

SEM micrographs of the wear track for the system composed by WC-Co/Al_2_O_3_ shows a completely different behaviour as illustrated in Figure 15. It is possible to observe a dense and homogeneous layer of oxides that covers the entire wear track. Figure 16 shows a micrograph of the border area between the wear track and unworn area while Figure 16b–f shows the respective chemical composition of the different elements obtained by EDS, confirming the presence of a dense layer of oxides.

The reason behind the greater stability exhibited by WC-Co’s friction curves and the lowest wear rate coefficient observed for the WC-Co/Al_2_O_3_ composite may be related to the formation of tribofilms/oxide layer. As the sliding between the disc and the counter-body occurs, debris is produced because of the extrusion of the binder phase as well as the fracture and fragmentation of WC grains, generating wear. A portion of the debris is retained in the wear track while the rest is accumulated on the periphery of the track. Debris from the WC and binder present on the track can agglomerate leading to the formation of a tribofilm. The layer formed, since it has different characteristics from the composite, can have a protective effect and influence both friction and resistance to consequent wear. The formation of tribofilms in the WC-Co composite during the tribological tests very likely justifies the greater stability of the friction curves as well as the lower wear coefficients. 

**Figure 17:**
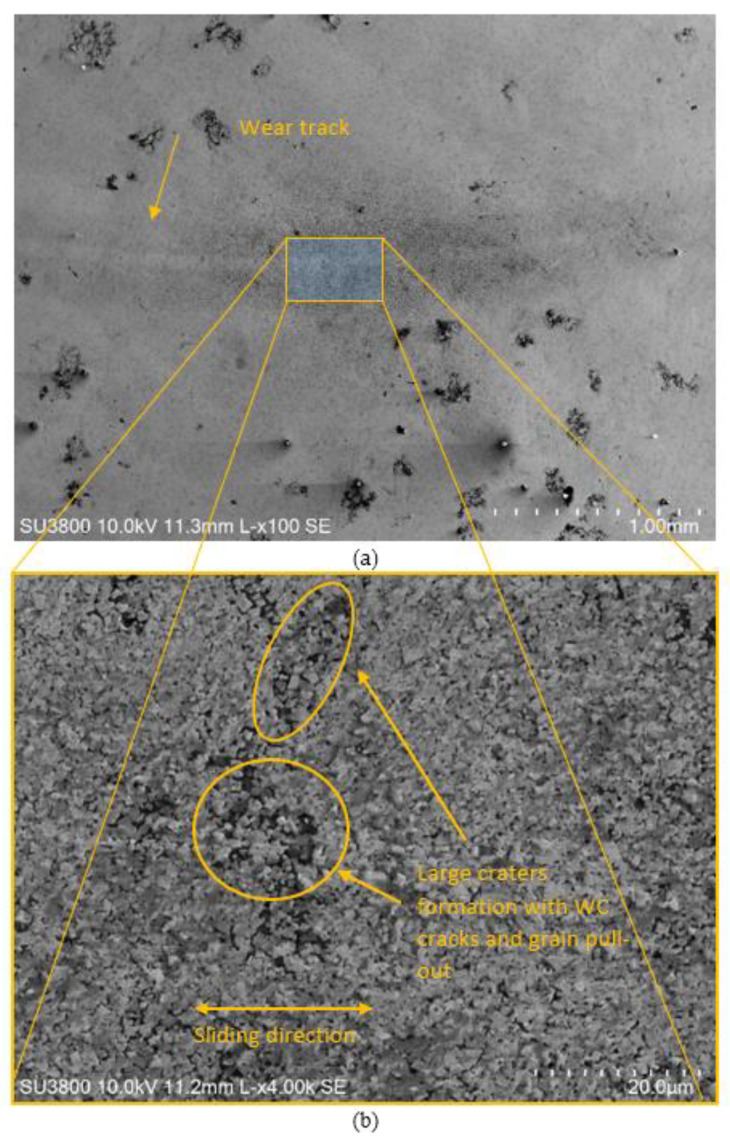
SEM micrographs of the wear track for the system WC-AISI 304/100Cr6 according to a magnification of: 100× (**a**), 4K× (**b**).

The same should not have occurred for the WC-AISI 304 composite, giving rise to unstable friction curves and higher wear coefficients as shown in Figure 11. By observing Figure 17 where micrographs of the wear track are shown for the WC-AISI 304/100Cr6 system, is possible to see that no tribofilm was formed and that large craters with WC cracks and grain pull-out occurred. The friction curves corresponding to some tests of the WC-AISI 304 composite are characterized by the presence of stable zones followed by peaks where the coefficient of friction increases abruptly, which goes down again and remains stable. This phenomenon may be related to the formation and destruction of tribolayers, in which the stable zones are associated with the formation of tribofilms while the peaks, where the friction coefficient rises, may indicate the pullout of the tribofilm previously formed. As far as the average coefficient is concerned, no clear and evident relationship was observed either between it and the friction curves or with the wear coefficients.

The third and final phase of discussion aims at the relationship between the mechanical properties and the tribological properties of the WC-based composites under study. The wear caused by the relative sliding between materials can be caused by adhesion, surface fatigue, tribochemical and abrasion mechanisms. Adhesion wear occurs when the roughness of the surfaces in contact forms an adhesive bond to each other, leading to plastic deformation, adhesion and subsequent fracture of a portion of the material. This phenomenon is related to the properties of hardness and fracture toughness. Otherwise, surface fatigue wear is based on fatigue processes associated with cyclical loads. This type of wear is characterized by the sequence: elastic deformation, plastic deformation, initiation and propagation of cracks and fractures. Thus, fracture toughness and modulus of elasticity are expected to play an important role in this type of wear. Abrasive wear occurs due to the penetration of hard particles, or roughness of a hard body on softer surfaces in relative motion and on the applied loading action. As hardness is considered a measure of resistance to penetration, this property is significant in this phenomenon. In addition, during sliding between surfaces, elastic and plastic deformations resulting from tribological contact are evidenced, which are associated with the elasticity and fracture toughness module. The most part of the times, several mechanisms occur at the same time, e.g.: matrix extrusion or binder removal, surface and subsurface fatigue and cracking of carbide grains, WC fracture and carbide pull-out and oxidation and tribofilm formation.

In this way, the main mechanical properties of WC-based composites are of ultimate importance: fracture toughness, hardness, and elastic modulus. Since in this work, values for the modulus of elasticity of the composites were not measured, the literature was used. C.M. Fernandes et al. [4] performed a mechanical characterization of the WC-10AISI 304 composite, estimating the modulus of elasticity at 513 ± 7 GPa. S. Okamoto et al. [34] investigated the mechanical properties of WC-Co composites with different grain sizes as well as contents in the binder phase. Based on the results presented by this author, the value of 550 GPa can be estimated for the WC-Co composite with 10 wt.% in a cobalt binding phase.

The analysis of the influence of mechanical properties on the wear of WC-based composites, assumes some complexity. However, by observing the wear rate values shown in Figure 11, it is possible to verify that, in general, the wear rate value increases with a decrease in fracture toughness values, more than with an increase in hardness. The minimum fracture toughness value of 7.8 MPa.m^1/2^ was obtained for the WC-AISI 304 (1542 HV30) specimen, which showed wear rates well above the WC-Co specimen (1491 HV30), which presented a value of 9.7 MPa.m^1/2^. It thus appears that fracture toughness is a factor that control the sliding wear behaviour of WC-based composites. Regarding the influence of the counter-body on the wear rate, there is evidence that a decrease in hardness changes the wear mechanism controlled by fracture to tribochemical. It is possible to conclude such behavior by analyzing the results obtained, considering that the pair WC-AISI 304/Al2O3 (3.760 × 10^−7^ mm^3^/N.m), showed a much higher wear rate than the pair WC-AISI 304/100Cr6 (2.854 × 10^−7^ mm^3^/N.m). This fact also helps to explain the transition from moderate to severe wear, in ceramic counter-bodies, for high severity values.

Comparison of results with previous investigations is not easy since there are no studies of evaluation of wear resistance to sliding wear with samples of WC-based composites with AISI 304 as a binder. However, it was possible to compare with some results by F. Djematene et al. [35] that performed a comparative study of the dry sliding wear that uses WC-10 wt.% (Co + Fe + Ni), varying the percentage of Co and Fe in the binder (Ni was set to 2 wt.%). The wear performance correlated inversely with the porosity and thus directly with the hardness with the lowest specific wear rate to be observed for the specimen using a Fe/Co ratio of 1. It should be noted that in this work the wear rate was an order of magnitude higher (1 × 10^−6^ mm^3^/Nm) than the results presented by us. This fact is easy to understand since our samples showed a densification of approximately 100%.

## 4. Conclusions

In the present study, mechanical characterization was carried out, as well as the tribological characterization of the WC-Co and WC-AISI 304 composites for two different counterbodies (100Cr6 and Al_2_O_3_), thus constituting four different tribological systems. the main conclusions drawn:

- For all tests, a rapid increase in the coefficient of friction was observed in the first sliding meters, corresponding to the running-in regime. However, the friction curves corresponding to the WC-Co composite tend to have a more stable behavior compared to the curves corresponding to the WC-AISI 304 composite.
- The system composed of the WC-AISI 304/100Cr6 tribological pair, corresponds to the lowest mean coefficient of friction, while the system consisting of the tribological pair WC-Co/100Cr6, corresponds the highest mean coefficient of friction. However the values did not vary much for the different systems.
- The wear volume tends to increase with increasing severity. The lowest wear coefficient corresponds to the WC-CO/Al_2_O_3_ tribological pair for which it was observed the formation of a tribofilms/oxide layer while the highest wear coefficient occurs for the system corresponding to the WC-AISI 304/Al_2_O_3_ tribological pair.
- In the present research work, it is possible to verify that, the wear rate coefficient increases with a decrease in fracture toughness values, more than with an increase in hardness (since the hardness assumes very similar values).
- As a general conclusion and attending to the wear rates behaviour, even if the WC-AISI 304 composite behaves slightly worse, is undoubtedly an alternative since the wear rates are of the same order of the reference composite, WC-Co.

## Figures and Tables

**Table 1:** Composition of WC-Co and WC-AISI 304 specimens.

	WC (wt.%)	Co (wt.%)	AISI 304 (wt.%)
WC-Co	90	10	-
WC-AISI 304	90	-	10

**Table 2:** Identification of the tribological systems under study.

Systems	Disc	Counterbody
WC-Co/100Cr6	WC-Co	100Cr6
WC-Co/Al_2_O_3_	WC-Co	Al_2_O_3_
WC-AISI 304/100Cr6	WC-AISI 304	100Cr6
WC-AISI 304/Al_2_O_3_	WC-AISI 304	Al_2_O_3_

**Table 3:** Hardness and fracture toughness of WC-Co and WC-AISI 304 specimens (the densification was approximately 100%).

Specimens	Hardness, HV30 (Kgf/mm^2^)		Fracture Toughness, K_IC_ (MPa·m^1/2^)		Grain Size, G (μm)	
	Average	STDEV	Average	STDEV	Average	STDEV
WC-Co	1491	36	9.7	1.1	2.7	0.4
WC-AISI 304	1542	18	7.8	0.3	2.1	0.2

**Table 4:** Conditions for the experimental tests corresponding to system WC-Co/100Cr6 (applied load corresponding to 20 N).

Test Number	N° Rotations	Time (s)	Sliding Distance (m)	Severity (N.m)
E1	36,000	10,800	2714	54,287
E2	50,000	12,500	3142	62,832
E3	72,000	18,000	4524	90,478
E4	100,000	25,000	6283	125,664

**Table 5:** Variation of the coefficient of friction for the system WC-Co/100Cr6.

Test Number	µ_med_	Δµ
E1	0.344	0.012
E2	0.414	0.021
E3	0.371	0.013
E4	0.341	0.027

**Table 6:** Variation of coefficients of friction for the systems: WC-Co/100Cr6, WC-Co/Al_2_O_3_, WC-AISI 304/100Cr6 and WC-AISI 304/Al_2_O_3_.

System	µ_med_	Δµ
WC-Co/100Cr6	0.368	0.029
WC-Co/Al_2_O_3_	0.353	0.046
WC-AISI 304/100Cr6	0.308	0.042
WC-AISI 304/Al_2_O_3_	0.357	0.038

**Table 7:** Wear coefficients of the counter bodies for the systems: WC-Co/100Cr6, WC-Co/Al_2_O_3_, WC-AISI 304/100Cr6 and WC-AISI 304/Al_2_O_3_ and corresponding increased number of times in relation to the wear coefficient of system WC-Co/Al_2_O_3_.

System	K_cb_ (mm^3^/N·m)	Increase
WC-Co/100Cr6	1.174 × 10^−8^	1.2 X
WC-Co/Al_2_O_3_	9.531 × 10^−9^	-
WC-AISI 304/100Cr6	9.380 × 10^−7^	98 X
WC-AISI 304/Al_2_O_3_	7.687 × 10^−8^	8 X

**Figure A1:**
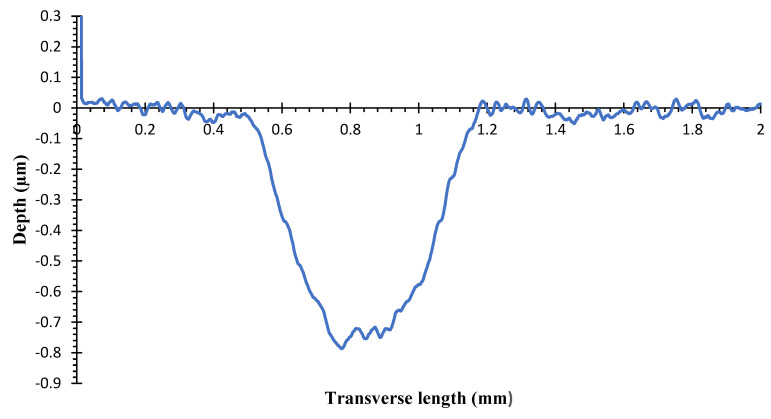
Example of cross-section 2D profile of a levelled and smoothed wear track.

**Figure A2:**
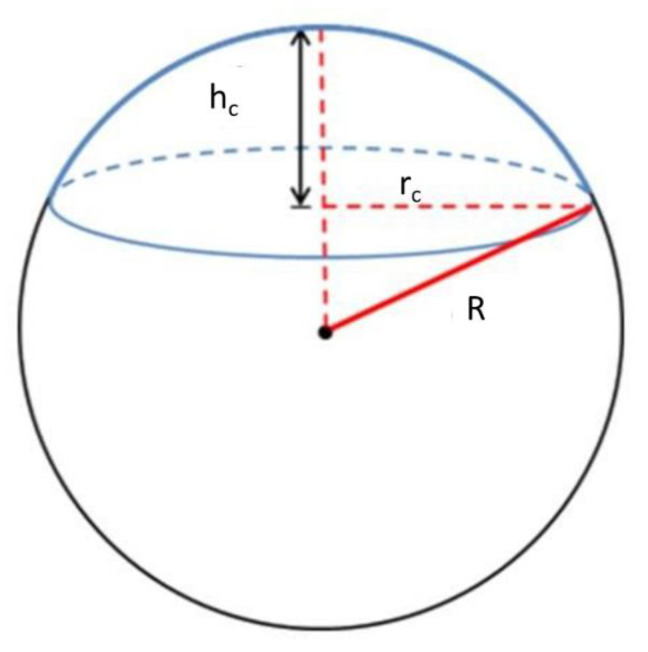
Representation of a spherical cap.

**Figure A3:**
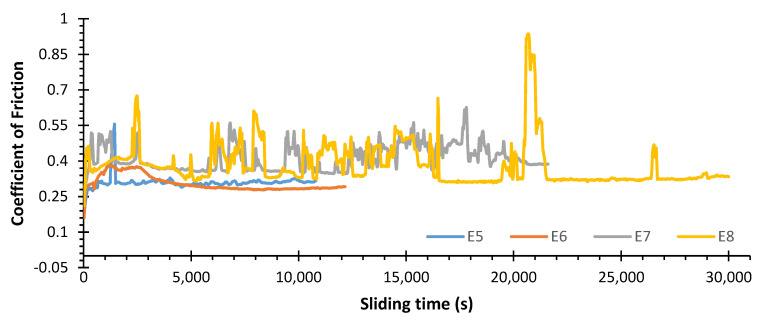
Coefficient of Friction (COF) curves of the tests performed for the system WC-Co/Al_2_O_3_.

**Figure A4:**
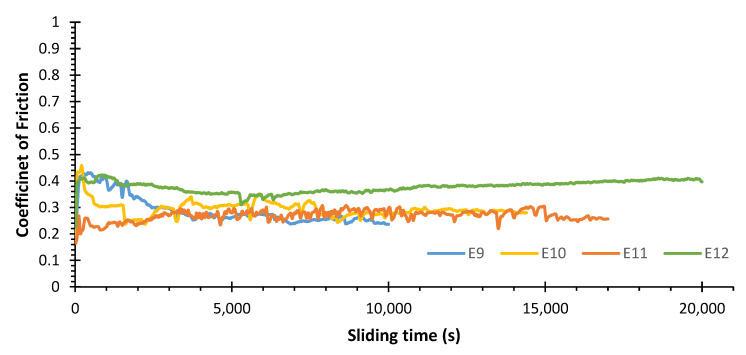
Coefficient of Friction (COF) curves of the tests performed for the system WC-AISI 304/100Cr6.

**Figure A5:**
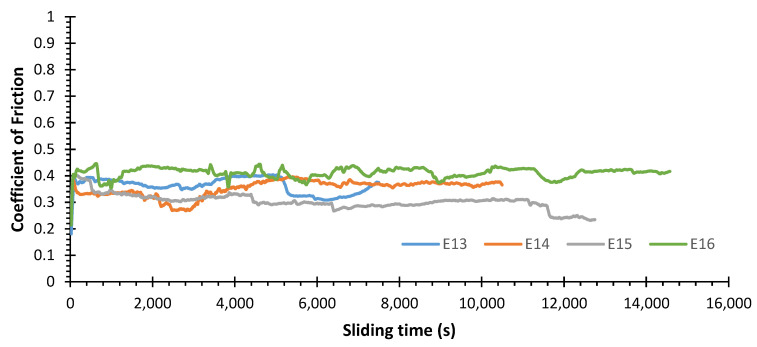
Coefficient of Friction (COF) curves of the tests performed for the system WC-AISI 304/Al_2_O_3_.

## Data Availability

The data presented in this study are available on request from the corresponding author. The data are not publicly available due to privacy.

## Appendix A

From the data collected on the tribometer, which provide the values of the tangential force and knowing that the normal force is constant in the tests, the instantaneous friction coefficient can be determined. This is given by the coefficient between the tangential force and the normal force verified at a given moment, as shown in Equation (A1):

(A1) $$\mu_{inst}=\frac{F_{T}}{F_{N}}$$

The average friction coefficient was also determined. For this, the arithmetic mean is applied to the set of instantaneous friction coefficients, as shown in Equation (A2):

(A2) $$\mu_{med}=\frac{\sum^{\;}\;\mu_{inst}}{n_{\mu_{inst}}\;}$$

This coefficient allows to obtain an overview of the friction coefficient verified for a given test. Finally, to complement the calculation of the average friction coefficient, its variation was determined by calculating the standard deviation, expressed by Equation (A3):

(A3) $$\sigma =\sqrt{\frac{\Sigma {\left(\mu_{inst}-\mu_{med}\right)}^{2}}{n_{\;\mu_{inst}}}}$$

To determine the wear volume of material in the WC-Co and WC-AISI 304 discs resulting from the tribological tests, the Mitutoyo SurfTest SJ-500 profilometer was used. The determination of the volume of the wear track was based on the two-dimensional profiles, transversal to the wear track, obtained through profilometry. However, for the subsequent calculation of the volume, an important simplification was assumed: the cross section of the wear track was considered constant along its perimeter. Thus, to minimize the error obtained by this simplification, for each track under analysis three 2D profiles were produced. A wear profile treated according to the described methods can be seen in Figure A1. It should be noted that the transverse area of the wear track corresponds to the area of the negative region present in the centre of the graph.

Taking this into account, the volume of the wear track is determined using the Equation (A4):

(A4) $$V_{p}=2\pi r_{p}A_{p}$$

where *r_p_* (mm) represents the radius of the wear track, corresponding to the distance between the centre of rotation of the disc and the centre of the sphere, used during the tribological test. *A_p_* (mm^2^) corresponds to the transverse area of the wear track. This area is determined by the numerical integration of the negative central region of the profile. Note that the area used for calculating the volume in Equation (A4), Ap, corresponds to the arithmetic mean of three areas obtained by treating the three 2D profiles created for each wear track. To determine the wear volume of the counter-body, an approximation of the body was made to the volume of a spherical shell. This volume is determined from the diameter of the circular wear mark left on the counter-body, which corresponds to the diameter of the base of the spherical cap. To minimize the approximation error, two diameters were measured, in two directions perpendicular to each other, using the Mitutoyo Toolmarker’s optical microscope. This volume is illustrated in blue in Figure A2.

The spherical cap height was determined using Equation (A5):

(A5) $$h_{c}=R-\sqrt{R^{2}-{\left(r_{c}\right)}^{2}}$$

where *h_c_* (mm) represents the height of the cap, *R* (mm) the radius of the sphere used as a counter body in the test and *r_c_* (mm) the radius of the base of the cap. The radius of the hubcap base was calculated from the arithmetic mean of the two measured diameters. The wear volume of the counter-body was calculated using Equation (A6):

(A6) $$V_{cb}=V_{c}=\frac{\pi }{3}{\left(h_{c}\right)}^{2}\left(3R-h_{c}\right)$$

The prediction of sliding wear is normally performed based on the equation proposed by Archard expressed in Equation (A7):

(A7) $$V=\frac{KSF_{N}}{H}$$

Subsequently, Czichos suggests the simplification presented in Equation (A8):

(A8) $$V=KSF_{N}$$

The modification proposed by Czichos is considerably simpler, which explains the fact that it is widely used. In this version, the hardness of the material is not considered. *V* (mm^3^) represents the wear volume, *K* (mm^3^.N^−1^.m^−1^) the wear coefficient, *S* (m) the sliding distance and ***F_N_*** (N) the normal force. Rearranging Equation (A8) to isolate the wear coefficient, Equation (A9) is obtained:

(A9) $$K=\frac{V}{SF_{N}}$$

The wear coefficient reflects the wear volume of a material for a given sliding distance and normal force. The sliding distance can be expressed by Equation (A10):

(A10) $$S=2\pi r_{p}N$$

where *S* represents the sliding distance for a given test, as a function of the wear track radius *r_p_* (m) and the number of test revolutions *N*.

To correlate the mentioned variables, for each constructed dispersion graph, a linear regression equation was applied where the slope of the linear regression line corresponds to the wear coefficient of the material.

## References

[1] Ortner H.M., Ettmayer P., Kolaska H. (2013). The history of the technological progress of hardmetals. Int. J. Refract. Met. Hard Mater..

[2] Fernandes C.M.S., Rocha A.F., Cardoso J.P.V., Bastos A., Soares E., Sacramento J., Ferreira M., Senos A. (2018). WC-stainless steel hardmetals. Int. J. Refract. Met. Hard Mater..

[3] Schmidt&Magnusson/Larsherberts Offset (2008). Understanding Cemented Carbides.

[4] Fernandes C., Vilhena L., Pinho C., Oliveira F., Soares E., Sacramento J., Senos A. (2014). Mechanical characterization of WC–10 wt% AISI 304 cemented carbides. Mater. Sci. Eng. A.

[5] Upadhyaya G.S. (2001). Materials science of cemented carbides—An overview. Mater. Des..

[6] Vilhena L.M., Fernandes C.M., Soares E., Sacramento J., Senos A.M.R., Ramalho A. (2016). Abrasive wear resistance of WC–Co and WC–AISI 304 composites by ball-cratering method. Wear.

[7] Lassner E., Schubert W.-D. (1999). Tungsten in Hardmetals. Tungsten.

[8] Prakash L. (2014). Fundamentals and General Applications of Hardmetals. Comprehensive Hard Materials.

[9] García J., Ciprés V.C., Blomqvist A., Kaplan B. (2019). Cemented carbide microstructures: A review. Int. J. Refract. Met. Hard Mater..

[10] Groover M.P. (2012). Fundamental of Modern Manufacturing Material, Processes, and System.

[11] IARC List of Classificaton—IARC Monographs on the Identification of Carcinogenic Hazards to Humans. https://monographs.iarc.fr/agents-classified-by-the-iarc/.

[12] ECHA Substance Information. https://echa.europa.eu/substance-information/-/substanceinfo/100.028.325.

[13] Global Energy Metals Corp. Cobalt. https://www.globalenergymetals.com/cobalt/cobalt-demand/.

[14] Tracey V. (1992). Nickel in hardmetals. Int. J. Refract. Met. Hard Mater..

[15] Uhrenius B. (1992). Phase Diagrams as a Tool for Production and Development of Cemented Carbides and Steels. Powder Met..

[16] Shon I.J., Jeong I.K., Ko I.Y., Doh J.M., Woo K.D. (2009). Sintering behavior and mechanical properties of WC-10Co, WC-10Ni and WC-10Fe hard materials produced by high-frequency induction heated sintering. Ceram. Int..

[17] Hanyaloglu C., Aksakal B., Bolton J. (2001). Production and indentation analysis of WC/Fe–Mn as an alternative to cobalt-bonded hardmetals. Mater. Charact..

[18] Pereira P., Vilhena L., Sacramento J., Senos A., Malheiros L., Ramalho A. (2021). Abrasive wear resistance of WC-based composites, produced with Co or Ni-rich binders. Wear.

[19] Roulon Z., Lay S., Missiaen J. (2020). Interface characteristics in cemented carbides with alternative binders. Int. J. Refract. Met. Hard Mater..

[20] Sun J., Zhao J., Gong F., Ni X., Li Z. (2019). Development and Application of WC-Based Alloys Bonded with Alternative Binder Phase. Crit. Rev. Solid State Mater. Sci..

[21] Ojo-Kupoluyi O.J., Tahir S.M., Baharudin B.T.H.T., Hanim M.A.A., Anuar M.S. (2017). Mechanical properties of WC-based hardmetals bonded with iron alloys—A review. Mater. Sci. Technol..

[22] Pittari J.J., Murdoch H., Kilczewski S.M., Hornbuckle B.C., Swab J.J., Darling K.A., Wright J.C. (2018). Sintering of tungsten carbide cermets with an iron-based ternary alloy binder: Processing and thermodynamic considerations. Int. J. Refract. Met. Hard Mater..

[23] Tarraste M., Kübarsepp J., Juhani K., Mere A., Viljus M. (2019). Effect of Carbon Stabilizing Elements on WC Cemented Carbides with Chromium Steel Binder. Mater. Sci..

[24] Chychko A., García J., Ciprés V.C., Holmström E., Blomqvist A. (2022). HV-KIC property charts of cemented carbides: A comprehensive data collection. Int. J. Refract. Met. Hard Mater..

[25] Wentzel E., Allen C. (1997). The erosion-corrosion resistance of tungsten-carbide hard metals. Int. J. Refract. Met. Hard Mater..

[26] Hochstrasser S., Latkoczy C., Virtanen S., Uggowitzer P., Schmutz P. (2007). Analytical Characterization of the Corrosion Mechanisms of WC-Co by Electrochemical Methods and Inductively-Coupled Plasma Mass Spectroscopy. Corros. Sci..

[27] Oliveira A., Bastos A., Fernandes C.M.S., Pinho C., Senos A., Soares E., Sacramento J., Zheludkevich M., Ferreira M. (2015). Corrosion behaviour of WC-10% AISI 304 cemented carbides. Corros. Sci..

[28] Chang S.H., Chen S.L. (2014). Characterization and properties of sintered WC-Co and WC-Ni-Fe hard metal alloys. J. Alloys Compd..

[29] Marques B., Fernandes C.M.S., Senos A. (2013). Sintering, microstructure and properties of WC-AISI304 powder composites. J. Alloys Compd..

[30] Rgp Balls Sfere in Acciaio al Cromo AISI 52100 100Cr6. https://www.rgpballs.com/it/sfere-in-acciaio-al-cromo-aisi-52100-100cr6/.

[31] CeramTec Oxide Ceramics—Aluminum Oxide. https://www.ceramtec.com/ceramic-materials/aluminum-oxide/#.

[32] Shetty D.K., Wright I.G., Mincer P.N., Clauer A.H. (1985). Indentation fracture of WC-Co cermets. J. Mater. Sci..

[33] Salguero J., Vazquez-Martinez J.M., Del Sol I., Batista M. (2018). Application of Pin-On-Disc Techniques for the Study of Tribological Interferences in the Dry Machining of A92024-T3 (Al–Cu) Alloys. Materials.

[34] Okamoto S., Nakazono Y., Otsuka K., Shimoitani Y., Takada J. (2005). Mechanical properties of WC/Co cemented carbide with larger WC grain size. Mater. Charact..

[35] Djematene F., Djerdjare B., Boukantar A., Rezzoug A., Abdi S., Daoud I. (2020). A comparative study of the dry sliding wear of WC-10wt.%(Co+Fe+Ni) cemented carbides pressureless sintered with different Fe/Co ratios. J. Asian Ceram. Soc..

